# Cross-Neutralization Potential of Native Human Papillomavirus N-Terminal L2 Epitopes

**DOI:** 10.1371/journal.pone.0016405

**Published:** 2011-02-08

**Authors:** Michael J. Conway, Linda Cruz, Samina Alam, Neil David Christensen, Craig Meyers

**Affiliations:** 1 Department of Microbiology and Immunology, The Pennsylvania State University College of Medicine, Hershey, Pennsylvania, United States of America; 2 Department of Pathology, The Pennsylvania State University College of Medicine, Hershey, Pennsylvania, United States of America; Karolinska Institutet, Sweden

## Abstract

**Background:**

Human papillomavirus (HPV) capsids are composed of 72 pentamers of the major capsid protein L1, and an unknown number of L2 minor capsid proteins. An N-terminal “external loop” of L2 contains cross-neutralizing epitopes, and native HPV16 virions extracted from 20-day-old organotypic tissues are neutralized by anti-HPV16 L2 antibodies but virus from 10-day-old cultures are not, suggesting that L2 epitopes are more exposed in mature, 20-day virions. This current study was undertaken to determine whether cross-neutralization of other HPV types is similarly dependent on time of harvest and to screen for the most effective cross-neutralizing epitope in native virions.

**Methodology and Principal Findings:**

Neutralization assays support that although HPV16 L2 epitopes were only exposed in 20-day virions, HPV31 or HPV18 epitopes behaved differently. Instead, HPV31 and HPV18 L2 epitopes were exposed in 10-day virions and remained so in 20-day virions. In contrast, presumably due to sequence divergence, HPV45 was not cross-neutralized by any of the anti-HPV16 L2 antibodies. We found that the most effective cross-neutralizing antibody was a polyclonal antibody named anti-P56/75 #1, which was raised against a peptide consisting of highly conserved HPV16 L2 amino acids 56 to 75.

**Conclusions and Significance:**

This is the first study to determine the susceptibility of multiple, native high-risk HPV types to neutralization by L2 antibodies. Multiple anti-L2 antibodies were able to cross-neutralize HPV16, HPV31, and HPV18. Only neutralization of HPV16 depended on the time of tissue harvest. These data should inform attempts to produce a second-generation, L2-based vaccine.

## Introduction

High-risk human Papillomavirus (HPV) virions are the etiologic agents of numerous anogenital and oropharyngeal cancers [1], [2]. Over 99% of cervical cancers are caused by persisent infections with high-risk HPVs which accounts for the second highest cancer burden in women worldwide next to breast cancer [1], [2]. HPV capsids contain a single, circular dsDNA genome of approximately 8 kb, which associates with histones to form a chromatin-like structure [3], [4]. The viral DNA is packaged within a nonenveloped, icosahedral capsid composed of 72 pentamers of the major capsid protein L1 and an unknown number of the minor capsid protein L2 [5], [6]. High resolution images of bovine papillomavirus 1 (BPV1) and HPV16 pseudovirions (PsV) suggest that the inner conical hollow of L1 pentamers can be occluded with a monomer of L2 [5], [6]. Importantly, an N-terminal “external loop” of L2 exists which can be the target of neutralizing and cross-neutralizing antibodies [7], [8], [9], [10]. It is unknown if the external loop threads through the center of L1 pentamers or between them [5], [9].

We showed previously that organotypic culture-derived HPV16 virions exploited a tissue-spanning redox gradient that facilitated assembly and maturation events in the context of the complete papillomavirus life cycle [11], [12]. Importantly, neutralization of HPV16 by anti-L2 antibody RG-1 (a.a. 17-36) depended on the maturation state of the virion [11], [12]. Virions extracted from 20-day-old tissue were neutralized more effectively than virions extracted from 10 or 15-day-old tissue, which suggested that L2 loops were externalized over time [11], [12]. Further, substitutions of conserved N-terminal L2 cysteines for serine abrogated effective neutralization of 20-day HPV16 native virions, lending support to the hypothesis that redox and differentiation-dependent conformational changes in L2 occur in the context of stratified and differentiated human tissue [11], [12].

The current study was undertaken to determine whether cross-neutralization of other HPV types is dependent on the time of tissue harvest and to determine the most effective cross-neutralization epitope in L2 from native virions. We show that although availability of HPV16 L2 epitopes for neutralization was maximal in 20-day virions, HPV31 or HPV18 epitopes were already maximally exposed at 10-days, and remained so in 20-day samples. In contrast, HPV45 was not cross-neutralized by any of the anti-HPV16 L2 antibodies presumably because of sequence divergence. The most effective cross-neutralizing antibody was a polyclonal antibody named anti-P56/75 #1. Anti-P56/75 #1 was raised against a peptide consisting of HPV16 L2 amino acids 56 to 75. This epitope was 100% similar and 90–100% identical in all HPV types tested. These data are the first to determine the susceptibility of native, high-risk HPV types to antibodies that recognize cross-neutralizing, N-terminal, L2 epitopes, and should inform attempts to produce a second-generation, L2-based vaccine.

## Results

### Establishment of HPV16, HPV31, HPV18, and HPV45 containing stable cell lines

Stable cell lines that can synthesize HPV16, HPV31, HPV18, and HPV45 organotypic culture-derived native virions were described previously by our lab [11], [13], [14], [15], [16]. Briefly, these cell lines were obtained by electroporating primary human foreskin keratinocytes (HFKs) with linearized HPV16, HPV18, and HPV45 full-length genomes and selecting immortalized stable cell lines [11], [14], [15], [16]. The CIN-612 9E cervical intraepithelial neoplasia type I biopsy-derived cell line was utilized for the production of HPV31 [13]. From these stable cell lines, organotypic cultures were grown for 10 or 20-days, at which point, crude viral preps (CVPs) were generated by dounce homogenization in phosphate buffer, followed by salt extraction of virions. As described previously, all CVPs were treated with benzonase to remove free or susceptible virus-associated genomes prior to performing additional experiments [11].

### RT-qPCR-based neutralization and cross-neutralization of 10 and 20-day HPV16 and HPV31 virions

**Figure 1:**
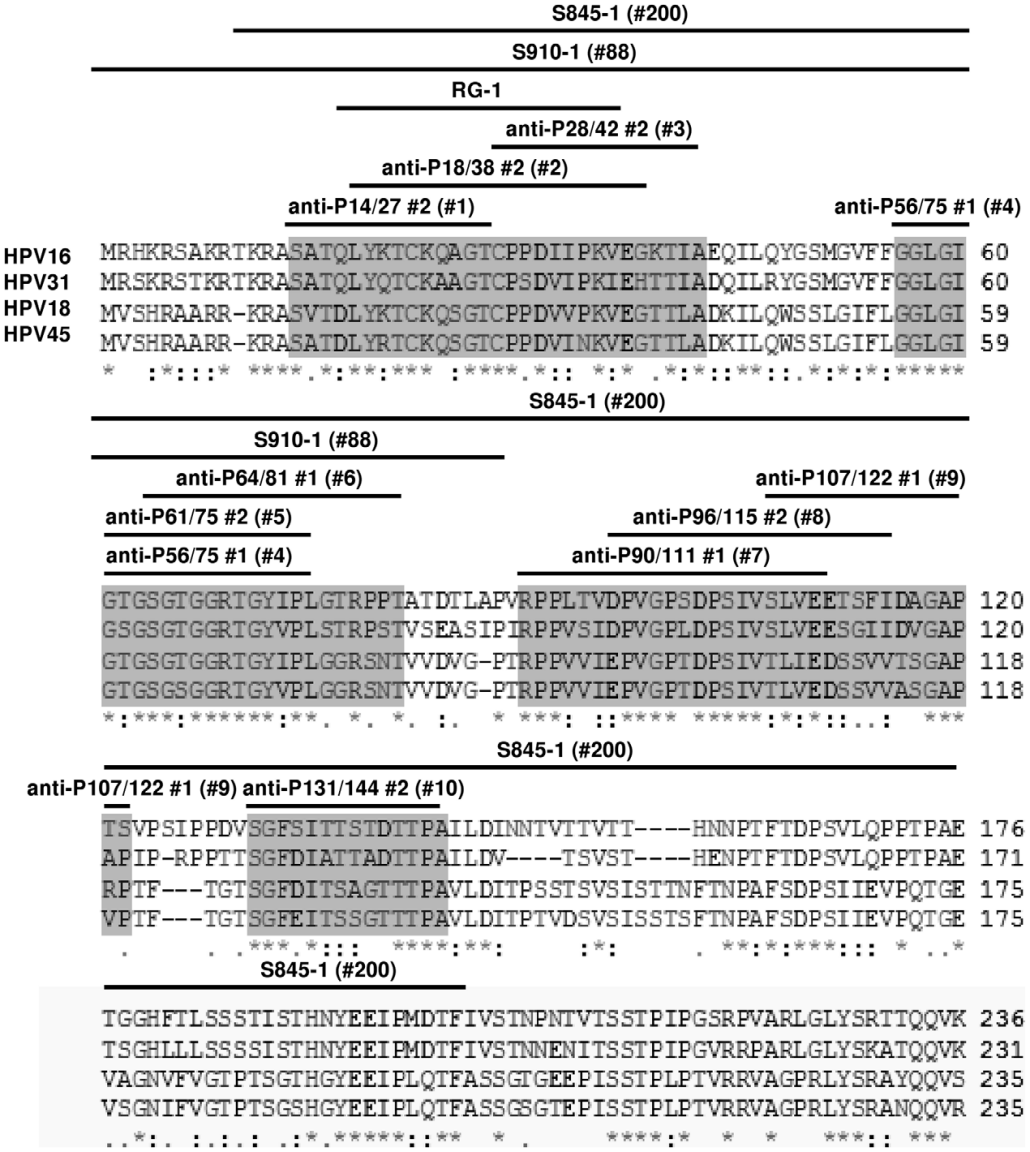
Alignment of HPV16, HPV31, HPV18, and HPV45 L2. The N-terminal 236 amino acids of HPV16 L2 were aligned against HPV31, HPV18, and HPV45 L2 using NCBI BLAST. Asterisks denote 100% amino acid identity. Colons denote conservation of amino acid properties. Periods denote conservation of some of the amino acids. White space denotes a lack of amino acid conservation. Grey shaded regions indicate stretches of N-terminal amino acids that are exposed as antigens on the capsid surface. Black bars highlight N-terminal L2 peptides that were used to raise the antibodies listed above them.

To compare the potential of the L2 external loop in the evolutionarily related HPV types HPV16 and HPV31 as a neutralization and cross-neutralization target, a panel of anti-HPV16 L2 external loop-targeting antibodies that recognize epitopes within amino acids 14–144 were obtained and utilized in RT-qPCR-based neutralization assays [7], [8], [10]. Primers used for qPCR-based assays can be seen in Tables S1 and . Table S3 and Fig. 1 list the origin of each antibody and their previously published neutralization and cross-neutralization titers against HPV16, HPV31, HPV18, and HPV45 pseudovirions (PsV). For all experiments, antibodies were used at a 1∶100 dilution for neutralization of approximately 10 vge/cell of HPV16 and 100 vge/cell of HPV31. 1∶100 dilutions of each antibody are usually the highest concentration reported for neutralization tests of PsV, quasivirions (QV), and organotypic culture-derived native virions [7], [8], [10], [12]. Even though 10-fold more vge/cell was used with HPV31 compared to HPV16, this allowed for normalization of RT-qPCR data, allowing amplification of nucleic acid at similar Ct values (data not shown).

**Figure 2:**
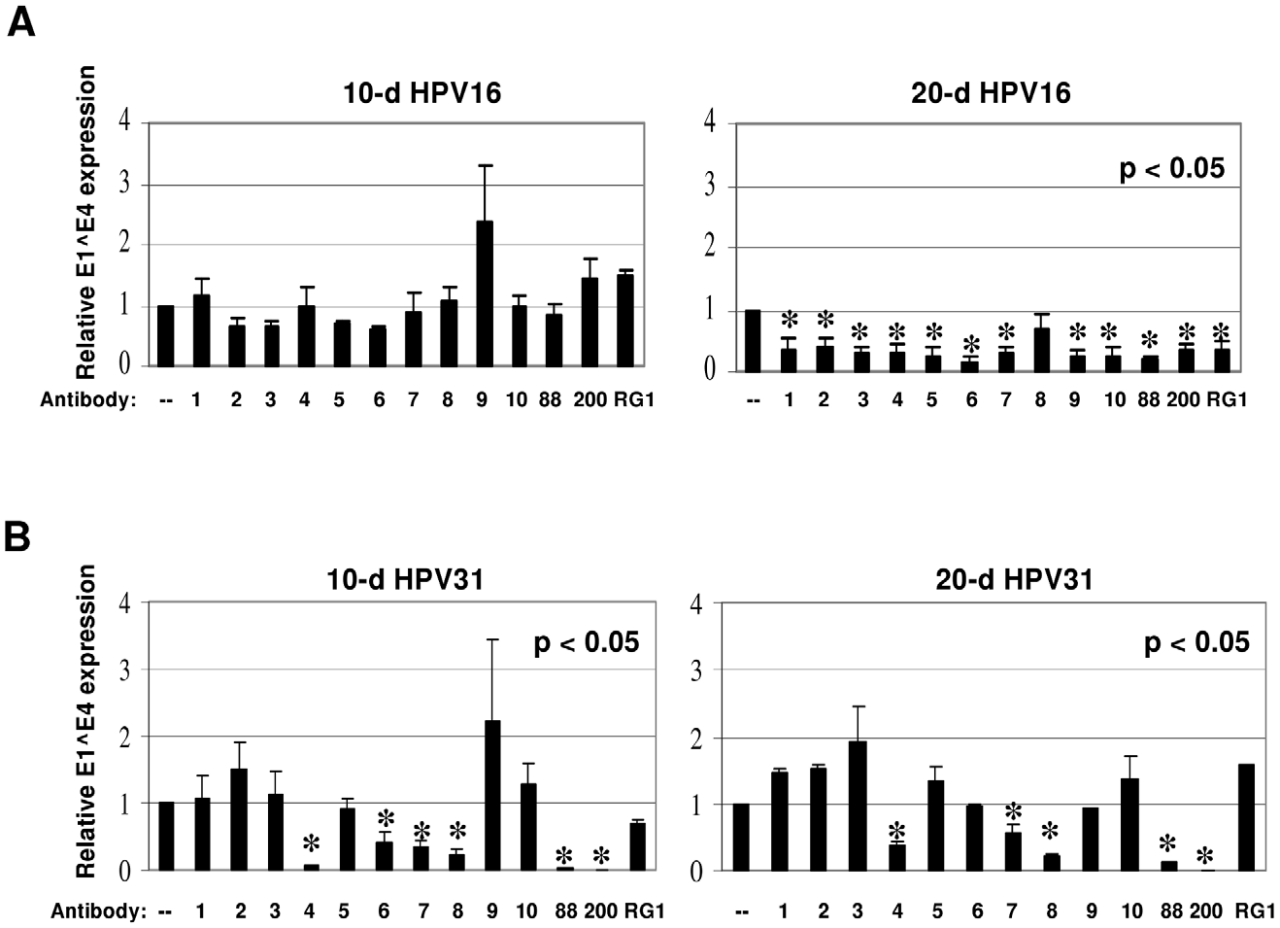
Neutralization of HPV16 (A) and cross-neutralization of HPV31 (B) CVPs with L2 antibodies. 50 µl of each 10 or 20-day CVP was diluted 1∶10 in HaCaT media with or without (–) a 1∶100 dilution of each antibody. Neutralization reactions were incubated for 1 hour at 37°C prior to infection of 5×10^6^ HaCaT cells. RNA was harvested and infectivity was assessed by measuring relative E1^E4 expression by duplex RT-qPCR. No antibody control values (–) were set to 1.0. Detailed information regarding the abbreviated antibodies #1-RG-1 can be seen in Table S3 and Fig. 1. All experiments were performed in triplicate and standard error of the mean was calculated.

Surprisingly, even though all antibodies were raised against either HPV16 L2 peptides or full-length HPV16 L2, none of these antibodies neutralized 10-day HPV16 (Fig. 2A). In contrast, 20-day HPV16 were neutralized by nearly all the antibodies, as each reduced E1^E4 expression about 60–80% compared to untreated controls (Fig. 2A). The exception was anti-P96/115 #2, which did not reduce E1^E4 expression (Fig. 2A).

The neutralization profile of HPV31 was very different from that of HPV16 (Fig 2B). Neutralization of 10-day HPV31 generated a profile very similar to that of 20-day HPV31 (Fig. 2B). HPV31 was not neutralized by anti-P14/27 #2, anti-P18/38 #2, anti-P28/42 #2, anti-P61/75 #2, anti-P107/122 #1, or anti-P131/144 #2 (Fig. 2B). However, several of the antibodies reduced E1^E4 expression as much as 95% and others to a lesser extent (Fig. 2B).

### RT-qPCR-based cross-neutralization of 10 and 20-day HPV18 and HPV45 virions

To assess the potential of the L2 external loop in the evolutionarily related HPV types, HPV18 and HPV45 as a cross-neutralization target, the panel of anti-HPV16 L2 external loop-targeting antibodies that recognize epitopes within amino acids 14–144 were utilized in RT-qPCR-based neutralization assays (Table S3) [7], [8], [10]. For all experiments, antibodies were utilized at a 1∶100 dilution with 3,000 vge/cell of HPV18, or 50 vge/cell of HPV45. Even though 60-fold more vge/cell was utilized with HPV18 compared to HPV45, this allowed for normalization of RT-qPCR data, allowing amplification of nucleic acid at similar Ct values (data not shown). This normalized infections with HPV16 and HPV31 as well.

**Figure 3:**
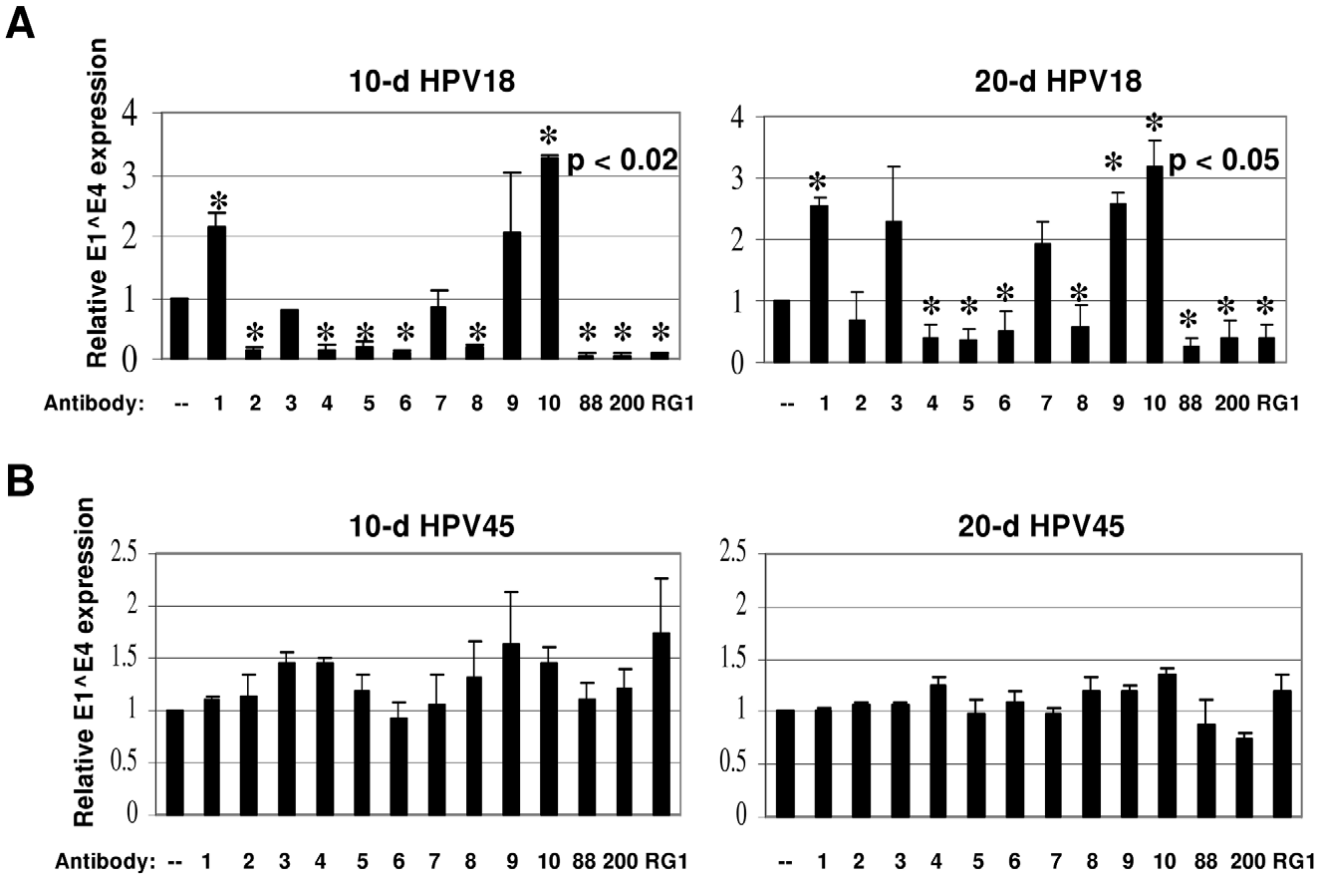
Cross-neutralization of HPV18 (A) and HPV45 (B) CVPs with L2 antibodies. 50 µl of each 10 or 20-day CVP was diluted 1∶10 in HaCaT media with or without (–) a 1∶100 dilution of each antibody. Neutralization reactions were incubated for 1 hour at 37°C prior to infection of 5×10^6^ HaCaT cells. RNA was harvested and infectivity was assessed by measuring relative E1^E4 expression by duplex RT-qPCR. No antibody control values (–) were set to 1.0. Detailed information regarding the abbreviated antibodies #1-RG-1 can be seen in Table S3 and Fig. 1. All experiments were performed in triplicate and standard error of the mean was calculated.

The neutralization profiles of 10 or 20-day HPV18 were very similar. Several of the antibodies reduced E1^E4 expression by 20–95% (Fig. 3A). However, significant differences in neutralizing activity were seen when neutralizing 10 compared to 20-day HPV18 virions with anti-P28/42 #2 and anti-P90/111 #1, making it difficult to make conclusions with these antibodies (Fig. 3A). We also noted significant increases in infectivity when HPV18 virions were pre-incubated with anti-P14/27 #2, anti-P107/122 #1, and anti-P131/144 #2.

The neutralization profile of HPV45 was very different from all of the other types tested in that it was resistant to neutralization at either time of harvest regardless of the antibody tested (Fig. 3B). We hypothesized that the inability to neutralize HPV45 was either due to sequence divergence, or conformational differences in L2 epitopes compared to HPV16 that hindered antibody binding. Although HPV45 was not neutralized, we found that the most effective cross-neutralizing antibody was anti-P56/75 #1, which neutralized 20-day HPV16 and cross-neutralized 10- and 20-day HPV31 and HPV18.

### Inefficient binding of anti-P56/75 #1 to HPV45 L2

Since HPV45 harvested at 10 and 20 days was resistant to cross-neutralization by all 13 antibodies tested (Fig. 3B), the percent identities and similarities of HPV31, HPV18, and HPV45 L2 epitopes were determined as compared to HPV16 L2 epitopes (Table 1). Generally, susceptibility to cross-neutralization correlated with the degree of conservation of a specific L2 epitope. For example, anti-P64/81#1 cross-neutralized HPV31 and HPV18, whose L2 epitopes share 83% identity, but anti-P64/81#1 didn't cross-neutralize HPV45 whose L2 epitope shares only 72% identity with HPV16 (Table 1). This analysis assumes that L2 epitopes are equally exposed on the surface of the capsid of each type and that antibody binds to L2 epitopes of each type to the same extent. The anti-P56/75#1 epitope is 90–100% conserved in HPV31, HPV18, and HPV45 compared to HPV16 (Table 1). Yet anti-P56/75#1, which cross-neutralized 10 and 20-day HPV31 and HPV18, failed to cross-neutralize 10 and 20-day HPV45. The anti-P56/75#1 epitope in HPV45 L2 differs only in two amino acid (i.e. T66S and I73V) than to HPV16 L2 (Fig. 1). Since the HPV31 antiP56/75#1 epitope also has the I73V substitution, the T66S substitution must have either singly or additively altered the immunogenicity of the HPV45 anti-P56/75#1 epitope (Fig. 1). Another possibility is that the anti-P56/75#1 epitope is inaccessible to antibody binding in the context of the complete HPV45 viral particle.

**Table 1:** Percent identities and similarities of HPV31, HPV18, and HPV45 L2 epitopes compared to HPV16 L2 epitopes.

		HPV31	HPV31	HPV18	HPV18	HPV45	HPV45
Antibody	Abbr^a^	PI^b^	PS^c^	PI	PS	PI	PS
anti-P14/27 #2	#1	86%	93%	79%	86%	79%	93%
anti-P18/38 #2	#2	71%	90%	***86%***	***100%***	81%	95%
anti-P28/42 #2	#3	67%	80%	73%	93%	73%	87%
anti-P56/75 #1	#4	***90%***	***100%***	***100%***	***100%***	90%	100%
anti-P61/75 #2	#5	87%	100%	***100%***	***100%***	87%	100%
anti-P64/81 #1	#6	***83%***	***89%***	***83%***	***83%***	72%	83%
anti-P90/111 #1	#7	***82%***	***95%***	64%	95%	68%	95%
anti-P96/115 #2	#8	***80%***	***85%***	***60%***	***95%***	65%	95%
anti-P107/122 #1	#9	63%	69%	38%	69%	44%	69%
anti-P131/144 #2	#10	71%	79%	64%	79%	71%	79%
S910-1	#88	***76%***	***86%***	***67%***	***80%***	60%	67%
S845-1	#200	***74%***	***84%***	***55%***	***73%***	***55%***	***73%***
RG-1	RG-1	75%	85%	***75%***	***90%***	70%	85%

a , Abbreviated names for antibodies for the purposes of figure simplification in this manuscript.

b , Percent identity measured the percent of identical residues in either HPV31, HPV18, or HPV45 L2 epitopes compared to HPV16 L2 epitopes in relation to the length of the epitope.

c , Percent similarity measured the percent of residues which share amino acid properties in either HPV31, HPV18, or HPV45 L2 epitopes compared to HPV16 L2 epitopes in relation to the length of the epitope.

• Bold and italicized values indicate the HPV types that are neutralized by the adjacent antibody.

**Figure 4:**
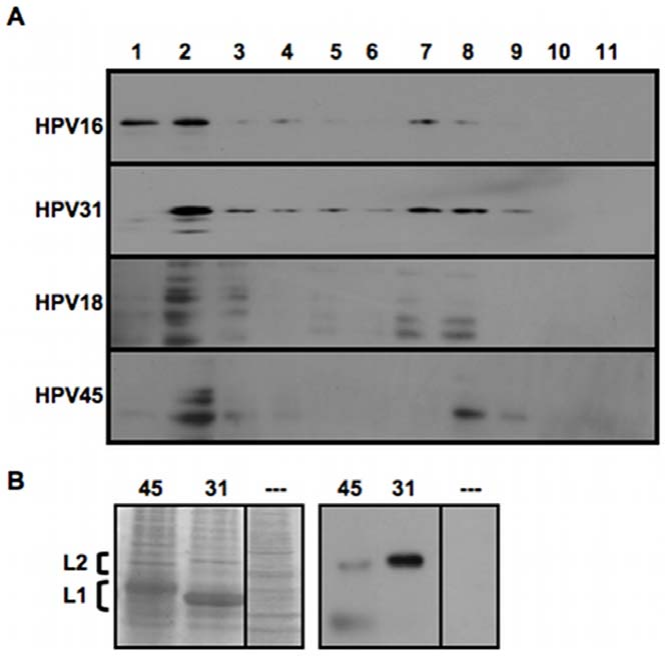
Western blot analysis of Opriprep-purified L1+L2 VLPs. (A) HPV16, HPV31, HPV18, and HPV45 VLPs were Optiprep-fractionated and fractions were assayed for L2 content by probing Western blots with anti-HPV16 L2 polyclonal antibody anti-P56/75 #1. (B) Side-by-side SDS-PAGE gels run with equivalent amounts of HPV45 and HPV31 L1+L2 VLPs from Optiprep fraction #8 were either Coomassie-stained, or Western blotted with anti-P56/75 #1. L2 and L1 bands are indicated by brackets (note the small size difference in L2 and the larger size difference in L1). A control lane that represents an SDS-PAGE gel of Optiprep-purified 293TT cell lysate without capsid protein was also included for the Coomassie stained samples (—).

To determine if the failure of anti-P56/75#1 to cross-neutralize HPV45 was due to the inability of the antibody to recognize a slightly altered HPV45 L2 epitope, Western blot analyses of reduced L1 and L2-containing HPV16, HPV31, HPV18, and HPV45 VLPs were performed using anti-P56/75#1 to detect L2 (Fig. 4A). As seen in Fig. 4A, anti-P56/75#1 successfully recognized HPV16, HPV31, HPV18, and HPV45 L2 linear epitopes.

However, equivalent levels of immunoreactivity were not observed after performing Western blot analysis on samples loaded with equal amounts of HPV31 and HPV45 L2 protein based on Coomassie Blue staining (Fig. 4B). Compared to HPV31 L2, there was a significant decrease in the binding of anti-P56/75#1 to HPV45 L2, suggesting that the single T66S substitution in the HPV45 anti-P56/75#1 epitope was sufficient to alter immunogenicity. In contrast, 10-day HPV45 was neutralized over 90% by 1∶100 dilutions of polyclonal antisera generated from HPV45 L1/L2 VLPs, supporting that the failed neutralization assays were due to the use of the less conserved HPV16 L2 antibodies (data not shown). These results suggest that the inability of anti-P56/75#1 to cross-neutralize HPV45 was due to lack of binding to the slightly altered HPV45 L2 linear epitope, rather than failure of the HPV45 L2 epitope to be conformationally accessible to antibody binding.

## Discussion

We showed previously that native virions exploit a tissue-spanning redox gradient, which facilitated assembly and/or maturation events in the context of the complete papillomavirus life cycle [11]. Importantly, neutralization of HPV16 by the anti-L2 antibody RG-1 was dependent on the maturation state of the virion. Virions extracted from 20-day-old tissue were neutralized more strongly than virions extracted from 10 or 15-day-old tissues, suggesting that the L2 loop was externalized over time [11]. The anti-HPV16 L2 antibodies anti-P14/27 #2 (a.a. 14–27), anti-P56/75 #1 (a.a. 56–75), and #S910-1 (a.a. 1–88) also strongly neutralized 20-day HPV16 but not 10-day HPV16 [12]. Here we extended those observations, using a panel of anti-HPV16 L2 “external loop” targeting antibodies to determine if the neutralization of other HPV types depended on the time of virus harvest. Both HPV31 and HPV18 extracted from either 10 or 20-day-old tissue were cross-neutralized similarly. However, regardless of the time of harvest, HPV45 was not cross-neutralized by any of the HPV16 L2 antibodies.

We reasoned that sequence divergence in L2 epitopes rather than the lack of proper display of those epitopes could explain the lack of cross-neutralization. The epitope most conserved in the two types is recognized by anti-P56/75 #1 and shares 90% percent identity and 100% percent similarity. Furthermore, this HPV45 epitope differs from the HPV31 epitope by only a single amino acid (i.e. T66S) and the anti-P56/75 #1 antibody cross-neutralized both 10 and 20-day HPV31. Anti-P56/75 #1 strongly reacted with the linear epitope of HPV31 but not HPV45. Thus, the single, very conservative, T66S substitution was sufficient to hinder recognition of HPV45 L2 by this antibody, consistent with our hypothesis that the sequence changes are responsible for the failure of at least some anti-HPV16 L2 antibodies to cross-react with HPV45. Even with its failure to cross-neutralize HPV45, we found that the most effective cross-neutralizing antibody was anti-P56/75 #1, which did neutralize and cross-neutralize HPV16, HPV31, and HPV18. Although anti-P56/75 #1 was unable to neutralize HPV45, a concatemer, designated L2 11–88×5, which is composed of the conserved N-terminal sequence of amino acids 11–88 from L2 proteins of the distantly related HPV types 1, 5, 6, 16, and 18 did induce antibodies in both mice and rabbits. These antibodies cross-neutralized HPV45 PsV [17], [18]. These data suggest that raising a multitype antibody response against L2 is an effective technique to cross-neutralize a large number of papillomavirus types.

We also compared previously published neutralization and cross-neutralization data for HPV PsV to our data for native virions derived from organotypic culture (Table S3) [7], [8], [10]. Significant differences were apparent (compare Figs. 2–3 to Table S3). These comparisons encompass many publications and experimental protocols, so the differences in neutralizing activity between PsV and native virions most likely arise from methodological considerations rather than from physical properties of the individual particles.

These experiments highlight the utility of N-terminal L2 epitopes in eliciting cross-neutralizing antibodies that can neutralize infectivity of virions synthesized in stratifying and differentiating human epithelial tissue. Many antibodies neutralized 20-day HPV16 and cross-neutralized 10 or 20-day HPV31 and HPV18 in crude viral preparations, an environment which represents the physiological state of natural infection much more so than fractions of gradient-purified virus. The neutralization of virus in crude viral preparations shows that the abundant proteinaceous material in the prep does not interfere with antibody reactivity against organotypic culture-derived native virions. In addition, the failure of antibodies to neutralize HPV16 should not be a serious obstacle to the development of future L2-based vaccines because 10-day HPV16 particles reside in the suprabasal compartment of stratifying and differentiating human epithelial tissue. Any transmission of these highly unstable particles would require severe damage to release them from deep within the tissue. Such extensive damage is likely a rare event. A more concerning finding was that significant increases in infectivity were observed when antibodies were pre-incubated with HPV18. At present, the mechanism that led to the increase in infectivity is unknown and should be thoroughly investigated.

## Materials and Methods

### Ethics Statement

The use of discarded human foreskin keratinocyte (HFK) tissues to develop cell lines for these studies was approved by the Institutional Review Board at the Pennsylvania State University College of Medicine and by the Institutional Review Board at Pinnacle Health Hospitals.

### Keratinocyte cultures, and electroporation

Primary human foreskin keratinocytes (HFKs) were isolated from newborn circumcision as described previously [13], [14], [15], [16]. Briefly, keratinocytes were grown in 154 medium (Cascade Biologics, Inc., Portland, OR) supplemented with Human Keratinocyte Growth Supplement Kit (Cascade Biologics, Inc.). Electroporations of HPV16, HPV18, and HPV45 in primary human foreskin keratinocytes has been described previously [13], [14], [15], [16]. Following electroporation, HPV-positive cell lines were selected via immortalization as compared to HFKs that were mock transfected. The HPV31-containing 9E cell line, obtained from a low-grade CINI cervical lesion, has been described previously [13].

### Organotypic “raft” cultures

Immortalized HFK lines which stably maintained episomal HPV16, HPV31, HPV18, and HPV45 DNA were grown in monolayer culture using E medium in the presence of mitomycin C-treated J2 3T3 feeder cells. Raft tissues were grown as previously described [13], [14], [15], [16]. Briefly, HPV16-containing HFK lines were seeded onto rat tail type-1 collagen matrices containing J2 3T3 feeder cells not treated with mitomycin C. After epithelial attachment to the collagen matrices and growth to confluence, matrices were lifted onto stainless steel grids. Once lifted to the air-liquid interface, epithelial raft cultures were fed by diffusion from underneath with E medium which lacked epidermal growth factor (EGF) and was supplemented with 20 mM 1,2-dioctanoyl-sn-glycerol (C8:0, Sigma Chemical, St. Louis, MO). Raft cultures were allowed to stratify and differentiate for 10 and 20 days.

### HPV isolation

For Optiprep fractionation, RT-PCR, RT-qPCR, and qPCR-based DNA encapsidation assays, 3-raft crude viral preps (CVPs) were prepared by dounce homogenization in 500 µl phosphate buffer (0.05 M Na-phosphate [pH 8.0], 2 mM MgCl_2_). Homogenizers were rinsed with 250 µl phosphate buffer. 1.5 µl (375 units) Benzonase (Sigma) was added to 750 µl of CVPs and incubated at 37°C for 1 hour. Samples were adjusted to 1 M NaCl by adding 130 µl ice cold 5 M NaCL. Then, samples were vortexed and centrifuged at 4°C for 10 minutes at 10,500 rpm in a microcentrifuge. Supernatants were stored at −20°C.

### Quantitative RT-PCR infectivity assays

HaCaT [19] cells were seeded at 50,000 cells/well in 24-well plates in Dulbecco's modified Eagle's medium supplemented with 10% fetal bovine serum, 2 mM glutamine, 1 mM pyruvate, 100 units/ml penicillin, and 100 µg/ml streptomycin and grown to approximately 70% confluence. 50 µl HPV16 crude viral preps (CVPs) were diluted with cell culture medium to a total volume of 0.5 ml. Medium was aspirated from HaCaT cells and 0.5 ml of diluted CVPs was added per well. One well on each plate received 0.5 ml of medium without virus as a negative control. The cells were incubated with the virus for 48 h at 37°C. mRNA was harvested with the SurePrep TrueTotal RNA Purification Kit (Fisher Scientific). DNA contamination of columns was determined to be insignificant as the optional on-column DNase-I treatment of extracted mRNA had no effect on downstream signal. Amplification of both the viral target and endogenous cellular control target was performed using a duplex format in 0.2 ml, 96-well PCR plates (BIO-RAD) with a total reaction volume of 25 µl. All reactions containing RNAs from virus-infected cells were performed in duplicate or triplicate. Reverse transcription and quantitative PCR were performed in the same closed tube with approximately 250 ng of total RNA per reaction using the Quantitect Probe RT-PCR Kit (Qiagen). HPV16, HPV31, HPV18, and HPV45 E1^E4 primers are listed in Table S1. All were used at final concentrations of 4 µM. Fluorogenic, dual-labeled, HPV16, HPV31, HPV18, and HPV45 probes are also listed in Table S1. All were utilized at a final concentration of 0.2 µM to detect E1^E4 DNA. Primers and probe were developed using Gene Link Software: OligoAnalyzer 1.2, and OligoExplorer 1.2. TBP primer sequences were obtained from those previously described [20]. All primers were synthesized by Integrated DNA Technologies (Coralville, IA). All QRT-PCR reactions were performed using the iQ5 (BIO-RAD). Cycling conditions were 50°C for 30 min (reverse transcription) and 95°C for 15 min, followed by 42 cycles of 94°C for 15 s and 54.5°C for 1 min. Amplification efficiencies of each primer set was 93% for E1^E4 and 97% for TBP. Relative quantities of viral target cDNA were determined using REST© software. For antibody-mediated neutralizations, 50 µl of crude viral prep was incubated for 1 hour at 37°C prior to infection of HaCat cells with a 1∶100 dilution of the anti-HPV16 L2 antibodies listed in Table S3. A 1∶100 dilution of a polyclonal antibody generated from HPV45 L1/L2 VLPs (#5158, 45 L1/L2) was also used against 10-day HPV45. The #5158, 45 L1/L2 antibody was a gift from Richard Roden.

### qPCR-based DNA encapsidation assay

To detect endonuclease-resistant genomes in crude viral preps (CVPs) or Optiprep fractions, only benzonase-treated CVPs were utilized so that all non-encapsidated genomes were degraded. To release all encapsidated viral genomes, 10 µl sonicated virus prep or 20 µl Optiprep fraction was added to 2 µl Proteinase K, 10 µl 10% SDS, and 2 µl pCMV-GFP (140 ng/µl) carrier DNA, and adjusted to 200 µl with Hirt buffer. Tubes were rotated at 37°C for 2 hours. Immediately, an equal amount of phenol-chloroform-isoamyl alcohol (25∶24∶1) was added and the DNA was extracted into the aqueous phase. An equal amount of chloroform was added to the aqueous phase and again the DNA was extracted into the aqueous phase. DNA was EtOH precipitated overnight at −20°C. After centrifugation, the DNA pellet was washed with 70% EtOH and resuspended in 20 µl TE overnight. To detect viral genomes or cellular DNA, a Qiagen Quantitect SYBR Green PCR kit was utilized. Amplification of the viral target was performed in 0.2 ml, 96-well PCR plates (BIO-RAD) in a total reaction volume of 25 µl. l µl of each endonuclease-resistant viral genome prep was analyzed in triplicate for each independent experiment. All primers were used at a final concentration of 0.3 µM. A list of HPV16, HPV31, HPV18, and HPV45 primers used to amplify a region in E2 can be seen in Table S2. Oligonucleotides were synthesized by Integrated DNA Technologies (Coralville, IA). A standard curve was generated by amplifying 1 µl aliquots of 10^4^, 10^3^, 10^2^, and 10^1^ serially-diluted pBSHPV16, pBSHPV31, pBSHPV18, or pBSHPV45 copy number controls. Acceptable R^2^ values for standard curves were at or above 0.99. A Bio-Rad iQ5 Multicolor Real-Time qPCR machine and software were utilized for PCR amplifications and subsequent data analysis. The PCR thermocycling profile was as follows: 15 min. hot-start at 95°C, followed by 40 cycles at 15 sec. at 94°C, 30 sec. at 52°C, and 30 sec. at 72°C. Data analysis commenced during the extension phase. Melt curve analyses were performed for all SYBR Green PCR amplifications to verify specificity of the reaction. Melt curves and first derivative melt curves were run immediately after the last PCR cycle. Melt curves were produced by plotting the fluorescence intensity against temperature as the temperature was increased from 60 to 95°C at 0.5°C/s. Calculation of the exact number of endonuclease-resistant viral genomes per 3-raft crude viral prep was determined by comparing experimental values to the number of actual genome copies within the serially-diluted copy number controls.

### 293TT cell-based VLP production

HPV VLPs were generated in 293TT cells as previously described [21]. Briefly, 293TT cells, grown to 90% confluence in T-150 flasks were cotransfected by using Lipofectamine 2000 with 25 mg each of p16L1h (L1 expression plasmid) and p16L2h (L2 expression plasmid) (7). Cells were split 1∶2 at 24 h posttransfection and harvested 48 h posttransfection. Cell pellets were resuspended in a total of 750 µl of phosphate-buffered saline (PBS). Cells were lysed by Dounce homogenization as in the HPV isolation step described below. MgCl_2_ was added to a final concentration of 2 mM. Lysates were then incubated overnight at 37°C to allow maturation (7). Unpackaged DNA was digested by adding 0.2% benzonase (Sigma) and incubating for 1 h at 37 C°. After digestion, NaCl was added to a final concentration of 1 M. Cellular debris was removed, and virus-containing supernatant was collected after centrifugation at 10,500 rpm.

### Optiprep purification of VLPs

Optiprep purification was performed as described previously [21]. Briefly, 27, 33, and 39% Optiprep gradients were produced by underlayering. Gradients were allowed to diffuse for 1 to 2 h at room temperature. Then, 600 µl of clarified benzonase-treated virus preps were layered on top of the gradient. Tubes were then centrifuged in a SW55 rotor (Beckman) at 234,000×*g* for 3.5 h at 16°C. After centrifugation, 11 500 µl fractions were carefully collected from the top of each tube.

### Immunoblot analysis

Aliquots from Optiprep fractions were boiled for 10 min in 6% 2 mercaptoethanol (2-ME) loading buffer and loaded onto 8 to 10% polyacrylamide gels. For total protein analysis on SDS-PAGE gels via Coomassie Simply Blue (Invitrogen) staining, manufacturer's instructions were followed. To detect L2, anti-HPV16 L2 polyclonal antibody P56/75 #1 was utilized at a dilution of 1∶4,000.

## Supporting Information

Table S1 **Primer and probe sequences for RT-qPCR infectivity assays.**
(PDF)

Table S2 **Primer sequences for DNA encapsidation assays.**
(PDF)

Table S3 **Characteristics of anti-HPV16 L2 external loop-targeting antibodies.**
(PDF)
