# Supplementary material for: Cross-Neutralization Potential of Native Human Papillomavirus N-Terminal L2 Epitopes
Source: PLoS One. 2011 Feb 8;6(2):e16405. doi: 10.1371/journal.pone.0016405 (PMC3035607; doi:10.1371/journal.pone.0016405)
Supplement: Table S1 — Primer and probe sequences for RT-qPCR infectivity assays. (PDF) [file pone.0016405.s001.pdf]

Table S1
Primer and probe sequences for RT-qPCR infectivity assays

mRNA targets	5' Primer	3'Primer	Probe
HPV16 E1^E4	5'GCT GAT CCT GCA AGC AAC GAA GTA TC3'	5'TTC TTC GGT GCC CAA GGC3'	5'(6-FAM) CCC GCC GCG ACC CAT ACC AAA GCC (BHQ-1)3'
HPV31 E1^E4	5'TGG CTG ATC CAG CAA GTG AC3'	5'AGG CGC AGG TTT TGG AAT TC3'	5'(6-FAM) CAA AGC TAC CAA CAG CCA ACA ACA CCA CCA C (BHQ-1)3'
HPV18 E1^E4	5'GGC TGA TCC AGA AAC CAG TGA C3'	5'CTG GCC GTA GGT CTT TGC GGT G3'	5'(6-FAM) CCT CAC CGT ATT CCA GCA CCG TGT CCG TG (BHQ-1)3'
HPV45 E1^E4	5'CCA GAA ACC AGT GAC GAC ACG GTA TCC3'	5'GTG CCG ACG GAT GCG GTT3'	5'(6-FAM) AGC TAC AAC ACG CCT CCA CGT CGA CCC (BHQ-1)3'
TBP	5'CAC GGC ACT GAT TTT CAG TTC T3'	5'TTC TTG CTG CCA GTC TGG ACT3'	5'(5-HEX) TGT GCA CAG GAG CCA AGA GTG AAG A (BHQ-1)3'
