# Supplementary material for: Cross-Neutralization Potential of Native Human Papillomavirus N-Terminal L2 Epitopes
Source: PLoS One. 2011 Feb 8;6(2):e16405. doi: 10.1371/journal.pone.0016405 (PMC3035607; doi:10.1371/journal.pone.0016405)
Supplement: Table S2 — Primer sequences for DNA encapsidation assays. (PDF) [file pone.0016405.s002.pdf]

Table S2

Primer sequences for DNA encapsidation assays

DNA target	5' Primer	3'Primer
HPV16 E2	5'CCATATAGACTATTGGAAACACATGCGCC3'	5'CGTTAGTTGCAGTTCAATTGCTTGTAATGC3'
HPV31 E2	5'CAGTATTAACCACCAGGTGGTGGTG3'	5'GTTCAAGACTTGTTTGCTGCATTGTCC3'
HPV18 E2	5'TCCGCTACTCAGCTTGTTAAACAG3'	5'CCCACGGACACGGTGC3'
HPV45 E2	5'GAAGATGCAGACACCGAAGGAATC3'	5'GGCACACCTGGTGGTTTAGTTTGG3'
