# Supplementary material for: Cross-Neutralization Potential of Native Human Papillomavirus N-Terminal L2 Epitopes
Source: PLoS One. 2011 Feb 8;6(2):e16405. doi: 10.1371/journal.pone.0016405 (PMC3035607; doi:10.1371/journal.pone.0016405)
Supplement: Table S3 — Characteristics of anti-HPV16 L2 external loop-targeting antibodies. (PDF) [file pone.0016405.s003.pdf]

Table S3

Characteristics of anti-HPV16 L2 external loop-targeting antibodies

Antibody[a]	Peptide/Immunogen	Abbr.[b]	Reference	16 PsV [c]	31 PsV [c]	18 PsV [c]	45 PsV[c]
anti-P14/27 #2	HPV16 L2 amino acids 14-27	#1	Kondo et al., 2007	***≤50***	<50	<50	N/A
anti-P18/38 #2	HPV16 L2 amino acids 18-38	#2	Kondo et al., 2007	400	<50	100	N/A
anti-P28/42 #2	HPV16 L2 amino acids 28-42	#3	Kondo et al., 2007	800	<50	<50	N/A
anti-P56/75 #1	HPV16 L2 amino acids 56-75	#4	Kondo et al., 2007	400	200	200	N/A
anti-P61/75 #2	HPV16 L2 amino acids 61-75	#5	Kondo et al., 2007	800	***100***	200	N/A
anti-P64/81 #1	HPV16 L2 amino acids 64-81	#6	Kondo et al., 2007	3200	***50***	400	N/A
anti-P90/111 #1	HPV16 L2 amino acids 90-111	#7	Kondo et al., 2007	200	***≤50***	<50	N/A
anti-P96/115 #2	HPV16 L2 amino acids 96-115	#8	Kondo et al., 2007	***400***	400	***≤50***	N/A
anti-P107/122 #1	HPV16 L2 amino acids 107-122	#9	Kondo et al., 2007	100	<50	<50	N/A
anti-P131/144 #2	HPV16 L2 amino acids 131-144	#10	Kondo et al., 2007	200	<50	<50	N/A
S910-1	HPV16 L2 amino acids 1-88	#88	Pastrana et al., 2005	3,080	260	150	N/A
S845-1	HPV16 L2 amino acids 11-200	#200	Gambhira et al., 2007	40,960	N/A	2,560	N/A
RG-1 monoclonal	HPV16 L2 amino acids 17-36	RG-1	Gambhira et al., 2007	1,280	--	N/A	--

a, #1 or #2 after antibody names designates which animal was immunized.

b, Abbreviated names for antibodies for the purposes of figure simplification in this manuscript.

c, Antibody titers as previously determined by HPV16, HPV31, HPV18, and HPV45 PsV-based neutralization assays. Underlined and italicized titers represent radical differences between PsV-based and organotypic culture-based neutralization assays (compare Table S3 to Fig. 1 A-B and Fig. 2 A-B).
